# First-in-human evidence of multidrug resistance reversal in solid tumors: a cohort analysis of carbon nanoparticles‑Fe(II) complex trials

**DOI:** 10.1186/s12885-026-16216-7

**Published:** 2026-06-05

**Authors:** Ping Xie, Yuanfang Huang, Yongsheng Wang, Huashan Shi, Yan Chen, Zehui Gou, Ling Lai, Qi Dang, Xian Wu, Sheng-Tao Yang, Xiaohai Tang

**Affiliations:** 1 Sichuan Enray Pharmaceutical Sciences Company, Chengdu, 610041 China; 2 Department of Biotherapy, Cancer Center, West China Hospital, Sichuan University, Chengdu, 610041 China; 3 Division of Thoracic Tumor Multimodality Treatment, Cancer Center, State Key Laboratory of Biotherapy, West China Hospital, Sichuan University, Chengdu, 610041 China; 4 Department of Medical Ultrasound, West China Hospital, Sichuan University, Chengdu, 610041 China; 5 Department of Oncology, Hubei Key Laboratory for Translational Research in Traditional Chinese Medicine, the Central Hospital of Enshi Tujia and Miao Autonomous Prefecture, Enshi, 445000 China; 6 Phase I Clinical Research Center, Shandong First Medical University Affiliated Tumor Hospital, Jinan, 250117 China; 7 School of Chemistry and Environment, Southwest Minzu University, Chengdu, 610041 China

**Keywords:** Carbon nanoparticles‑Fe(II) complex, Ferroptosis, Multidrug resistance, Clinical trials, Nanomedicine

## Abstract

**Background:**

Multidrug resistance (MDR) is a major barrier to treat relapsed solid tumors. Carbon nanoparticles‑Fe(II) complex (CNSI-Fe) is composed of carbon nanoparticles and Fe²⁺ ions, which directly deliver Fe^2+^ into tumor cells to induce ferroptosis. In this cohort analysis from a Phase I trial, we aim to evaluate the safety, imaging response, survival outcomes, and potential restoration of sensitivity to systemic therapies.

**Methods:**

Nineteen patients in a Phase I study (NCT06048367) received the intratumoral injection of CNSI-Fe. The primary outcomes were safety and tolerability; secondary outcomes included radiographic response assessed by revised RECIST v1.1, tumor biology evaluated by necrosis and tumor growth rate (TGR), and long-term survival. All patients were followed after withdrawal from the study therapy.

**Results:**

CNSI-Fe demonstrated an acceptable safety profile. CNSI-Fe consistently induced marked central necrosis on imaging despite minimal revised RECIST v1.1 shrinkage. Four patients (4/19, 21.1%) with heavily pretreated, progressive disease (ovarian serous carcinoma, pancreatic adenocarcinoma, myoepithelioma-like tumor of the vulvar region, and thyroid carcinoma) achieved long-term survival for 28.9–36.2 months post CNSI-Fe injection, accompanied by restored response to chemotherapies or targeted agents to which they were previously resistant (e.g., resensitization-restored sensitivity, hypersensitization-enhanced sensitivity). These four patients (4/19, 21.1%) demonstrated renewed sensitivity to subsequent systemic therapies (e.g., paclitaxel, carboplatin, bevacizumab, irinotecan liposome, and lenvatinib).

**Conclusions:**

This case series provides preliminary clinical evidence that CNSI-Fe could weaken MDR and resensitize solid tumors to systemic therapy. These findings warrant biomarker-integrated expansion cohorts and Phase II trials of CNSI-Fe-based combinations with radiotherapy, chemotherapy, targeted therapy, and immunotherapy.

**Trial registration:**

The clinical data were derived from a completed prospective, single-arm, dose-escalation first-in-human trial (The Center for Drug Evaluation of the National Medical Products Administration, Registration No.: CTR20222235, Approval date: September 1, 2022; ClinicalTrials.gov Identifier: NCT06048367, First submitted date: August 24, 2023; RRID: SCR_002309).

**Supplementary Information:**

The online version contains supplementary material available at 10.1186/s12885-026-16216-7.

## Background

The treatment of metastatic solid tumors, particularly in patients who have developed acquired resistance following multiple lines of systemic therapy, remains a major challenge in clinical oncology [1, 2]. Although advancements have been made with traditional cytotoxic chemotherapy, targeted therapy, and immune checkpoint inhibitors, drug resistance and tumor heterogeneity often lead to treatment failure and disease progression [3, 4]. Therefore, there is a clear need for novel therapeutic strategies capable of overcoming multidrug resistance (MDR). However, clinical evidence for MDR reversal remains scare.

Ferroptosis is an iron-dependent, lipid peroxidation-driven form of regulated cell death [5, 6]. Preclinical studies demonstrated that ferroptosis effectively eliminated tumor resistance to apoptosis and stem-like properties [7, 8]. Damage-associated molecular patterns (DAMPs) released during ferroptosis remodeled the tumor immune microenvironment, shifting it from immunosuppressive to immune-supportive state [9, 10]. Despite these encouraging phenomena, the existing ferroptosis-inducing agents face poor bioavailability and lack of tumor-specific delivery, hindering their clinical translation [11, 12]. In particular, the ferroptosis-induced resensitization still await validation in first-in-human trials.

Carbon nanoparticles‑Fe(II) complex (CNSI-Fe) is composed of carbon nanoparticles and Fe²⁺ ions, which directly deliver Fe^2+^ into tumor cells upon intratumoral injection [13, 14]. Unlike other ferroptosis inducers that rely on systemic administration and the inhibition of SLC7A11 or GPX4, CNSI-Fe provides localized Fe²⁺ delivery into tumor tissue directly, thus simultaneously minimizing the systemic toxicity and maximizing intratumoral ferroptotic effect [14, 15]. Preclinical studies indicated that CNSI-Fe aroused strong ferroptosis [14, 16], and might also remodel the tumor immune microenvironment [15].

Of note, the spontaneous resensitization during drug holidays has been documented [17]. Herein, we hypothesized that CNSI-Fe treatment might have similar resensitization effect. Currently, a single-arm, dose-escalation first-in-human Phase I trial of CNSI-Fe has been conducted. We only enrolled the patients with documented disease progression prior to CNSI-Fe treatment. In this analysis of 19 patients from that trial, we aimed to test the hypothesis by checking the imaging response, long-term survival outcomes, and resensitization or hypersensitization to subsequent systemic therapies after CNSI-Fe injection. The implication to the ferroptosis-based therapy of CNSI-Fe in clinical applications is discussed.

## Methods

### Study design and patient sources

This was a single-arm, dose-escalation pilot study following a “3 + 3” design. The sample size was determined by empirical dose-escalation rules and feasibility considerations. Dose levels included 30, 60, 90, 120, and 150 mg, with 3, 4, 6, 3 and 3 patients assigned to each level, respectively. Key inclusion criteria were: (1) advanced solid tumor with progression after standard therapy, at least one measurable lesion amenable to intratumoral injection; (2) Eastern Cooperative Oncology Group (ECOG) performance status ≤ 1; (3) adequate hematologic and organ function. Key exclusion criteria were: (1) iron metabolism disorders; (2) risk of hollow organ perforation at the intended injection site; (3) local skin abnormalities interfering with drug administration. This analysis included all enrolled and treated patients from that trial (*N* = 19, the detailed treatments, safety assessment, and examinations for patients were listed in Supplementary text S1 and Table S1). Upon examining typical imaging characteristics and long-term survival outcomes, four cases exhibited post-treatment resensitization or hypersensitization.

### Imaging assessment

The tumor response was assessed using computed tomography (CT, Defintion AS, Siemens, Germany). Scans were performed at baseline and serially following treatment initiation. Intratumoral necrosis was checked by CT and tumor size was measured (CT measurement software: United Imaging, FULL-UIHClientProxy-net40-R001.0.3.1122872, ). The efficacy of CNSI-Fe for injected lesions was evaluated according to revised Response Evaluation Criteria in Solid Tumors (RECIST) guideline (v1.1). The tumor growth rate (TGR) was calculated for the periods before and after treatment [18]. TGR was calculated using a previously validated formula [18]:

$$\:\mathrm{T}\mathrm{G}\mathrm{R}=100\times\:\left[\mathrm{e}\mathrm{x}\mathrm{p}\right(\frac{3\times\:log(D2/D1)}{t})-1](\%/month)$$

where *D*_1_ and *D*_2_ are the sum of the longest diameters of the injected lesion at the first and second CT scans, and *t* is the time interval between the two scans expressed as months. Pre-treatment TGR was derived from historical imaging data available prior to study enrollment.

### Survival follow-up and analysis

All patients were followed for survival until death, loss to follow-up, or the data analysis cut-off date (December 10, 2025). Overall survival (OS) was defined as the time from the first administration of CNSI-Fe to the occurrence of any of the following events: (1) death; (2) the last known date the patient was alive (for those lost to follow-up); (3) the data analysis cut-off date. At the cut-off date, 8 patients remained alive and were under continued follow-up. Overall efficacy of CNSI-Fe was assessed according to RECIST v1.1. The relationship between overall efficacy and survival was analyzed, and subgroup analyses of patient survival were performed.

### Analysis of resensitization or hypersensitization cases

For the purpose of this analysis, the following operational definitions were pre-specified. Both resensitization and hypersensitization required: (1) documented disease progression prior to CNSI-Fe administration; and (2) subsequent achievement of partial response (PR) or complete response (CR) per revised RECIST v1.1 at the injected site after CNSI-Fe injection. Resensitization additionally required: (3) the response occurred following re-administration of the same or similar therapeutic agent to which the patient had previously progressed. Hypersensitization additionally required: (3) achievement of overall survival > 24 months after CNSI-Fe injection followed by systemic therapy. Four of the 19 patients met the criteria of resensitization or hypersensitization, whose detailed clinical data were collected, including encompassing demographic characteristics, complete treatment history (with particular focus on prior regimens to which resistance had developed), specifics of CNSI-Fe treatment (cycles and doses), radiological images before and after the resensitization or hypersensitization event, as well as subsequent treatment responses and survival data. Descriptive statistics were employed to summarize their clinical profiles. Furthermore, an individualized longitudinal analysis was performed by integrating each patient’s treatment timeline with imaging responses.

### Statistical analysis

The responses were scored by an experimenter blinded to injection condition and experimental cohort. Paired t-test was used to calculate the change in TGR (post-treatment minus pre-treatment) using IBM SPSS Statistics 26, with results presented as mean, 95% confidence interval (CI) and *p*-value. Survival analysis was performed using the Kaplan-Meier (KM) method (IBM SPSS Statistics 26). Results are reported as mean and median survival times with their corresponding 95% CI, along with 1-year and 2-year OS rates. For imaging assessments, patients with missing imaging data were excluded from the respective imaging analyses, but they were counted in the safety and survival analyses. Specifically, TGR was calculable in 14 of 19 patients, while 5 patients were excluded from TGR analysis due to incomplete imaging data. For efficacy assessment, one patient was excluded due to poor physical condition preventing post-treatment imaging, resulting in 18 evaluable patients. This was an open-label Phase I trial, therefore, blinding of patients and investigators was not feasible. To minimize bias, the following measures were taken: (1) for each patient, all serial imaging evaluations were performed on the same anatomical plane to ensure consistent lesion measurement; (2) the primary safety endpoint was based on objective Common Terminology Criteria for Adverse Events (CTCAE).

## Results

### Baseline patient characteristics

A total of 19 patients were enrolled. Baseline characteristics, including age, tumor type, and chronic comorbidities, are summarized in Table 1.

**Table 1:** Baseline patient characteristics (*n* = 19)

Number	Age (years)	Tumor type	Chronic conditions*
T01001	54	Ovarian serous adenocarcinoma	Hypothyroidism; Hyperglycemia; Hyperlipidemia; Hypertension
T01002	60	Lung squamous cell carcinoma	Hyperglycemia; Chronic gastritis; Chronic obstructive pulmonary disease (emphysema, chronic bronchitis); Fatty liver disease
T01003	52	Papillary thyroid carcinoma	/
T01004	64	Breast lobular ductal carcinoma	/
T01005	59	Pancreatic adenocarcinoma	Hypercholesterolemia; Liver cirrhosis; Pulmonary emphysema
T02001	69	Vulvar squamous cell carcinoma	Type 2 diabetes mellitus; Hyperlipidemia; Hypertension; Fatty liver disease; Bilateral lower extremity atherosclerotic plaques
T02002	53	MELTVR	Hypertension; Renal insufficiency
T01006	55	Pancreatic carcinoma	Hypertension
T01007	50	Liposarcoma	/
T01009	60	Squamous cell carcinoma of the buccal mucosa	Hypercholesterolemia; Benign prostatic hyperplasia
T01010	73	Sarcoma of the left mandible	Hypertension; Hypercholesterolemia
T01011	68	Liposarcoma	Benign prostatic hyperplasia
T03001	69	Fasciculated sarcoma	Hypertension; Hypercholesterolemia
T01012	53	Squamous cell carcinoma of the nasopharynx	Aortic sclerosis
T01013	44	Fasciculated sarcoma	Hyperlipidemia
T01014	55	Adenocarcinoma of the right nasal cavity	Hypothyroidism
T01015	55	Lung adenocarcinoma	Bronchial asthma; Reflux esophagitis; Cerebral infarction; Immunodeficiency; Chronic hepatitis C; Chronic gastritis
T01016	54	Breast infiltrating ductal carcinoma	Hypercholesterolemia
T01017	56	Lung adenocarcinoma	Hyperlipidemia

*Only typical chronic conditions requiring long-term management (e.g., hypertension, hypercholesterolemia, hypothyroidism) are listed. Incidental benign imaging findings (e.g., pulmonary nodules, renal cysts, hepatic cysts, pulmonary bullae) are not included as chronic comorbidities

/ No data; MELTVR, myoepithelioma-like tumor of the vulvar region

### Overview of safety

Among the 19 patients, a total of 307 adverse events (AEs) occurred, with 39 grade ≥ 3 AEs in 14 patients and 19 serious adverse events (SAEs) in 8 patients. In the full cohort, the most common AEs were injection site pain, elevated blood pressure, transient elevated serum iron, and anemia. Among the four patients with long-term survival and evidence of resensitization or hypersensitization, the grade ≥ 3 AEs included elevated blood pressure (2 patients), and hypersensitivity reaction with injection site pain (1 patient). The hypersensitivity reaction was an SAE. No treatment-related deaths were observed. Regarding dose exploration, no dose-limiting toxicity (DLT) was observed except one in the 90 mg dose group. Therefore, the highest administered dose was set at 150 mg, and the maximum tolerated dose (MTD) was not reached.

### Imaging characteristics

**Fig. 1:**
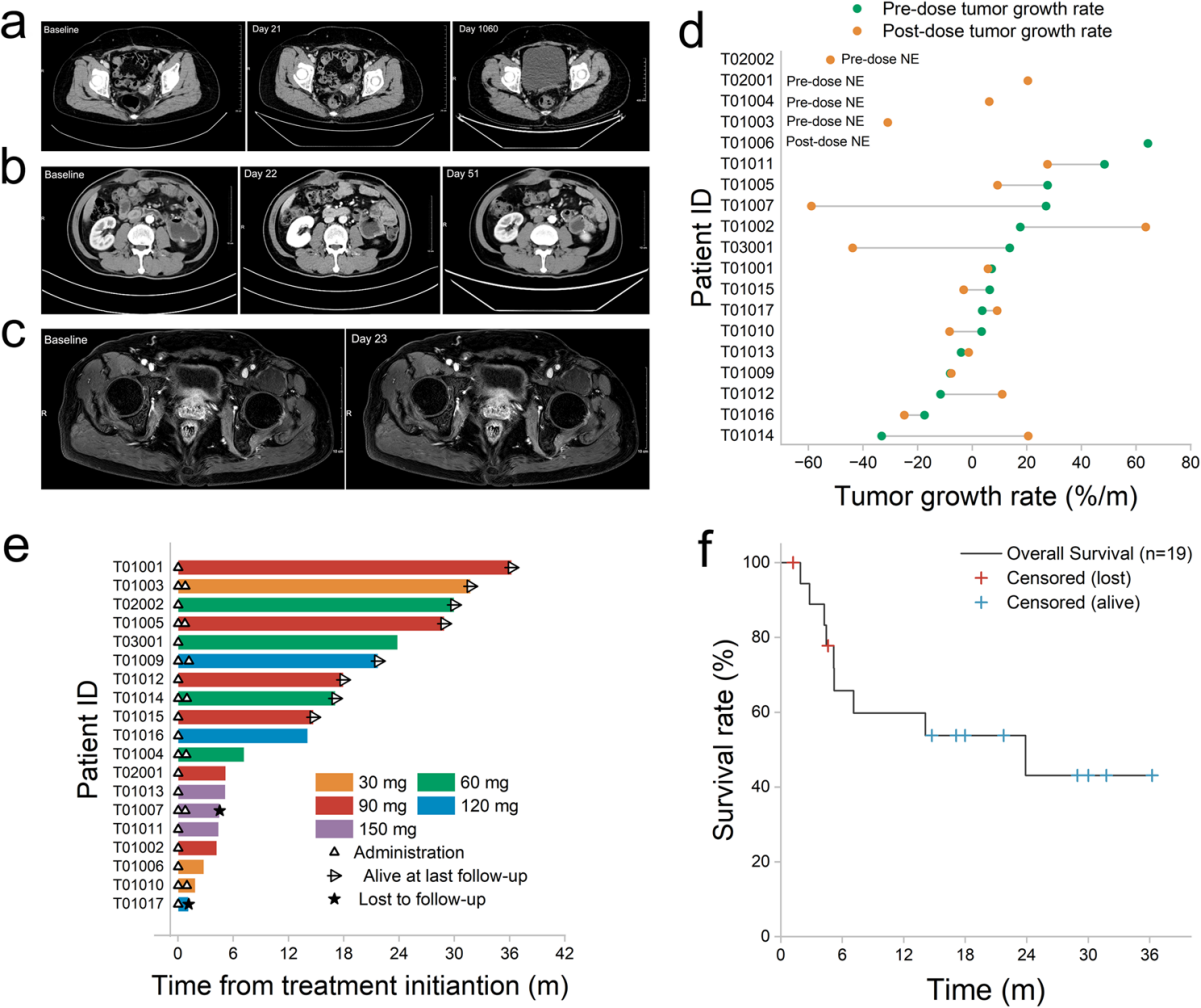
CT images, TGR diagrams and survival time diagrams of patients from Phase I clinical trials. **a**-**c** CT images of ovarian serous adenocarcinoma (**a**), pancreatic adenocarcinoma (**b**), and fasciculated sarcoma (**c**). **d** Paired TGR comparison before and after CNSI-Fe treatment (*n* = 14). Mean change: -5.85%/month (95% CI: -26.62 to 14.92, *p* = 0.554, paired t-test). **e** OS time for each patient. **f** KM curve of OS (median OS: 23.9 months, 95% CI: 0–50.6 months; 1-year OS rate: 59.8%; 2-year OS rate: 43.1%)

During tumor assessment imaging, a central necrosis pattern was observed at the injection site of CNSI-Fe, which was a rapidly emerging non-enhancing central area with a thickened viable rim. Representative CT images of three patients are shown in Fig. 1, including ovarian serous adenocarcinoma (T01001), pancreatic adenocarcinoma (T01005), and fasciculated sarcoma (T03001). These three patients exhibited tumor necrosis with efficacy assessments of stable disease (SD) post CNSI-Fe treatment. Notably, the patient with ovarian serous adenocarcinoma (T01001) received subsequent treatments of paclitaxel + carboplatin -/+ bevacizumab and bevacizumab + niraparib tosylate capsules after study discontinuation. During the 1060 days follow-up, the lesion of CNSI-Fe injection disappeared in CT images, and the subsequent treatments achieved Complete Response (CR).

### TGR analysis

Paired pre- and post-treatment TGR comparisons were available for 14 patients (Fig. 1d&S1), despite four lost their imaging results before enrollment and one patient was of poor physical condition for imaging post-treatment. The median pre-treatment TGR was 4.99%/month (range: -33.24 to 48.38%/month), which decreased to a median of 2.19%/month post-treatment (range: -58.97 to 63.51%/month). The mean TGR declined from 5.71%/month to -0.14%/month. The mean change in TGR (post-treatment minus pre-treatment) was − 5.85%/month (95% CI: -26.62 to 14.92%/month, *p* = 0.554). Eight of 14 evaluable patients (57.14%) exhibited a decrease in TGR, with notable reductions observed in certain cases (e.g., T03001, T01007).

### Survival outcomes

**Fig. 2:**
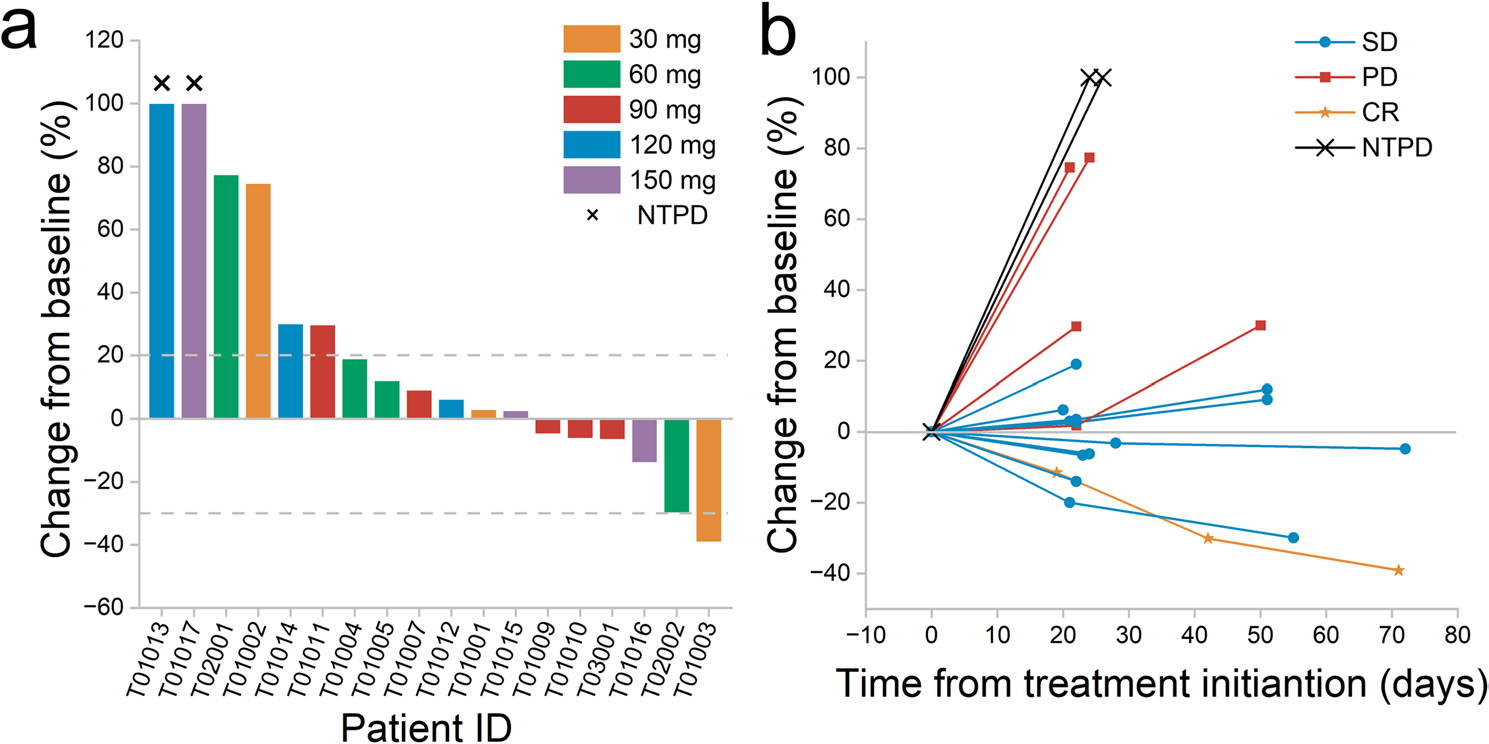
Overall efficacy in 18 patients (Among the 19 patients who received the treatment, one was not evaluated for efficacy due to poor physical condition, leaving 18 patients assessable for treatment response). **a** Waterfall plot showing the overall efficacy for 18 patients at the time of treatment discontinuation. Each bar represents one patient. **b** Spider plot illustrating the overall efficacy for the same 18 patients. Each line represents an individual patient. The “×” symbol indicates that the patient was assessed as having PD due to progression of non-target lesions (NTPD). For visualization purposes in the figure, this assessment was assigned a value of + 100%. SD, stable disease; PD, progressive disease; CR, complete response; NTPD, progression of non-target lesions

Among 19 treated patients, 9 patients died, 8 remained alive and 2 were lost to follow-up. Specific OS durations and KM survival curve are shown in Fig. 1e&f. Analysis using the KM method yielded a mean OS of 20.8 months (95% CI: 13.7 to 27.9 months) and a median OS of 23.9 months (95% CI: 0 to 50.6 months) for the cohort. The estimated 1-year and 2-year OS rates were 59.8% and 43.1%, respectively. Among 19 treated patients, 18 were evaluable for overall efficacy, as one patient was excluded from assessment due to poor physical condition. Individual patient-level overall efficacy data as shown in Fig. 2. Further subgroup analyses suggested that survival was significantly longer in patients achieving SD/CR, or with a largest target lesion/injected lesion < 5 cm, and showed a favorable trend in those with target-only, single, or ≤ 3 lesions (Figures S2-S7). Of particular importance, 4 patients achieved long-term survival ranging from 28.9 to 36.2 months, who were diagnosed with ovarian serous carcinoma, pancreatic adenocarcinoma, myoepithelioma-like tumors of the vulvar region (MELTVR), and papillary thyroid carcinoma, respectively.

### Resensitization or hypersensitization cases

In this study, resensitization or hypersensitization was observed in 4 patients (4/19, 21.1%). These 4 patients had previously received 2 to 7 lines of prior therapies (Table 1). After CNSI-Fe treatment and study discontinuation, all 4 patients demonstrated unexpectedly favorable responses to subsequent systemic therapies after CNSI-Fe treatment (Table 2). The remaining 15 patients (15/19, 78.9%), including those with early death, are detailed in the Supplementary text S2 and Tables S2&S3 (Table 3).

**Table 2:** Baseline characteristics and prior therapies

Number	Tumor type	Stage	Prior therapies and therapeutic effects		
			Time before enrollment (month)	Treatment	Efficacy
T01001	Ovarian serous adenocarcinoma	IVB	≈ 45	Liposomal paclitaxel + Lobaplatin	PR
			≈ 41	Paclitaxel + Carboplatin	/
			36^+^	Surgical resection^*^	/
			≈ 36	Paclitaxel + Carboplatin	PD
			25^+^	Gemcitabine	PD
			≈ 23	Etoposide + Bevacizumab	PD
			8^+^	PD-1 antibody	PD
T01003	Papillary thyroid carcinoma	I (high risk)	99^+^	Surgical resection^*^	/
			95^+^	Radionuclide 131 iodine	/
			≈ 48	Surgical resection^#^	/
			7^+^	Surgical resection^#^	PD
T01005	Pancreatic adenocarcinoma	IV	4^+^	Surgical resection^*^	/
			3^+^	Surgical resection^*^	/
			2^+^	Paclitaxel (albumin-bound type) + Gemcitabine	PD
T02002	MELTVR	IIIB	4^+^	Surgical resection^*^	/
			3^+^	Radiotherapy	PD

*PR* partial response, *PD* progressive disease, *MELTVR* myoepithelioma-like tumor of the vulvar region

* Tumor Resection

# Lymph Node Dissection

/ No data

**Table 3:** Summary of disease control and resensitization/hypersensitization

Number	Tumor type	Efficacy*	Subsequent treatment and therapeutic effect			Resensitization/ Hypersensitization	OS (month)	Survive
			Time after first administration (month)	Treatment	Efficacy*			
T01001	Ovarian serous adenocarcinoma	SD (+ 4.7%)	7^+^	Paclitaxel + Carboplatin	CR	Resensitization	36^+^	Yes
			7^+^	Paclitaxel + Carboplatin + Bevacizumab				
			12^+^	Bevacizumab+Niraparib mesylate				
T01003	Papillary thyroid carcinoma	CR (-39.1%)	≈ 13	Lenvatinib	CR	Hypersensitization	31^+^	Yes
			≈ 17	Surgical resection^#^				
T01005	Pancreatic adenocarcinoma	SD (+ 12.0%)	2	Irinotecan liposome+Oxaliplatin +(5-Fluorouracil+Calcium folinate)	PR	Hypersensitization	28^+^	Yes
			2^+^	Radiotherapy + Tegafur/ Gimeracil/Oteracil Potassium				
			8^+^	Tegafur/Gimeracil/Oteracil Potassium				
			≈ 19	Irinotecan liposome + Oxaliplatin + (5-Fluorouracil + Calcium folinate)				
T02002	MELTVR	CR (-69.4%)	1^+^	Albumin paclitaxel+ Cisplatin+Bevacizumab	CR	Hypersensitization	30	Yes
			8^+^	Bevacizumab				

*SD* stable disease, *CR* complete response, *PR* partial response, *MELTVR* myoepithelioma-like tumor of the vulvar region

* Efficacy of the injected lesion

# Lymph Node Dissection

#### Case 1: stage IVB ovarian serous carcinoma with documented resistance to platinum-taxane chemotherapy

During CNSI-Fe treatment, the best radiographic response was SD (+ 4.7%) of the injected lesion. Seven months post-treatment, the patient received chemotherapy comprising six cycles in total (one cycle of paclitaxel plus carboplatin, and five cycles of paclitaxel, carboplatin, plus bevacizumab), and followed by maintenance therapy [bevacizumab combined with a poly ADP-ribose polymerase inhibitor (PARP) inhibitor]. Follow-up CT scan indicated the overall therapy achieved a CR. At last follow-up, the patient remained alive with an OS exceeding 36 months.

#### Case 2: advanced papillary thyroid carcinoma with cervical lymph node metastases and a history of multiple surgical resections and radioactive iodine failure

CNSI-Fe treatment achieved a CR of the injected lesion. Thirteen months later, lenvatinib therapy was initiated. Follow-up CT imaging revealed no lymph node identification at the injection site, representing a second confirmed CR, followed by surgical resection of residual disease. The patient remained alive with an OS exceeding 31 months.

#### Case 3: advanced pancreatic adenocarcinoma with progression after multiple lines of chemotherapy

CNSI-Fe treatment achieved a SD (+ 12.0%) of the injected lesion. Subsequent systemic treatments, including liposomal irinotecan-based regimens and oral S-1, resulted in Partial Response (PR) and prolonged disease control. The patient remained alive with an OS exceeded 28 months.

#### Case 4: MELTVR with failure of multiple therapies

CNSI-Fe treatment achieved a CR of the injected lesion. After one month, the patient initiated systemic therapy with nab-paclitaxel, cisplatin, and bevacizumab, followed by continued bevacizumab monotherapy. These therapy remained CR, and the patient remained alive with an OS of 30 months.

### Subsequent Phase Ib/IIa clinical study

A Phase Ib/IIa expansion study (CTR20243192) was initiated following the Phase I trial. As of the data cutoff date, 45 patients have been enrolled, of whom 30 were evaluable for efficacy. Preliminary analysis indicated that the post‑treatment radiographic features of tumors in patients receiving CNSI-Fe were consistent with the Phase I findings, predominantly manifesting as central tumor necrosis. Among the evaluable patients, those with disease control (SD, PR and CR) had longer OS compared to those with progressive disease (PD). Furthermore, three additional cases of resensitization (lung adenocarcinoma, neuroendocrine tumor and melanoma) were observed in this study.

## Discussion

This study reports the exploratory clinical results of the novel ferroptosis inducer CNSI-Fe in patients with advanced solid tumors. All enrolled patients, who had failed multiple prior lines of therapy, achieved a median OS of 23.9 months with 1-year and 2-year OS rates of 59.8% and 43.1%. Notably, distinct clinical phenomenon of post-treatment resensitization or hypersensitization was identified in 4 patients (4/19, 21.1%).

CNSI-Fe might have exerted its antitumor effect by inducing ferroptosis in our study. The central necrosis of tumor might be attributed to the characteristic membrane disintegration caused by lipid peroxidation in ferroptosis [19], but patient-level ferroptosis biomarkers were not measured. It should be noted that the imaging necrosis is a non-specific finding and does not necessarily correlate with clinical benefit [20]. In our study, some patients showed substantial necrosis but minimal tumor shrinkage. This discrepancy could be explained by: (1) post-necrotic inflammation (edema/fibrosis) masking volume reduction [21]; (2) coagulative necrosis preserving tissue architecture without volume loss [22]; (3) early assessment timing before absorption completes [23]; and (4) tumor heterogeneity (viable regions continuing to grow) [24]. Importantly, imaging necrosis alone is not equivalent to clinical benefit [20]. Therefore, we reported necrosis as an exploratory imaging indicator rather than a primary efficacy endpoint. Future studies should incorporate functional imaging (e.g., PET-CT) to better elucidate its relationship with clinical outcomes [25].

The overall downward trend in TGR, particularly its conversion to negative values in some patients, suggested a potential kinetic signal of bioactivity [18]. However, the change did not reach statistical significance, likely due to the small sample size and data heterogeneity. The mean TGR declined from 5.71%/month to -0.14%/month, suggesting a deceleration in tumor growth and tumor shrinkage. These results suggested that CNSI-Fe might have decelerated tumor growth in a subset of patients. Our observation of TGR as a kinetic biomarker was consistent with recent studies validating TGR for prognostic stratification and treatment response monitoring in solid tumors [26, 27]. Ferroptosis might bypass or reverse certain mechanisms underlying traditional therapy resistance, offering a theoretical basis for understanding its potential resensitization or hypersensitization effects [7, 9, 28]. The survival benefit might not only stem from the immediate cytotoxicity of CNSI-Fe, but also likely achieved through the induction of ferroptosis and remodeling of the tumor microenvironment, which resensitized or hypersensitized patients to subsequent therapies. This benefit appeared more pronounced in patients who achieved SD/CR or had smaller lesions (< 5 cm), while a trend was also seen in those with fewer baseline lesions, indicating that lower tumor burden may predict better outcomes. However, these findings are limited by the small sample size (*n* = 19) and require validation in larger prospective cohorts.

The resensitization or hypersensitization of patients represents the most translational finding of our study. These findings were based on only four patients and remain hypothesis-generating, and some alternative explanations should be considered. First, natural disease variability may explain some observed changes. Tumor growth and regression can exhibit spontaneous fluctuations even without therapeutic intervention, particularly in indolent or heterogeneous tumors [29]. Second, tumor heterogeneity may contribute to the observed restoration of drug sensitivity [30]. Advanced tumors often contain distinct subclones with differential innate sensitivity [31]. Relatively sensitive subclones may expand after resistant clones are eliminated by prior therapies, mimicking “resensitization” without true treatment-induced reversal. Third, treatment sequencing and timing effects cannot be excluded. Patients had received multiple prior therapies, and delayed responses or long washout periods could lead to regained sensitivity before CNSI-Fe administration, independent of the study intervention [32]. Fourth, chance and sampling error are possible given the small sample size (*n* = 19) and lack of a control group. Finally, potential confounding from subsequent therapies (e.g., chemotherapy, targeted therapy) administered after CNSI-Fe cannot be excluded. These may have contributed to the observed survival outcomes independently of CNSI-Fe. Despite these alternative explanations, we believed that the spontaneous recovery was unlikely in our cases. Although spontaneous recovery of tumor sensitivity has been documented in the literature [17], all patients had documented disease progression on prior therapy before receiving CNSI-Fe, and the resensitization or hypersensitization phenomenon occurred only after CNSI-Fe injection followed by re-administration of chemotherapeutic or targeted agents. Therefore, we hypothesized that CNSI-Fe might directly mediate this effect, despite this hypothesis requires further validation.

**Fig. 3:**
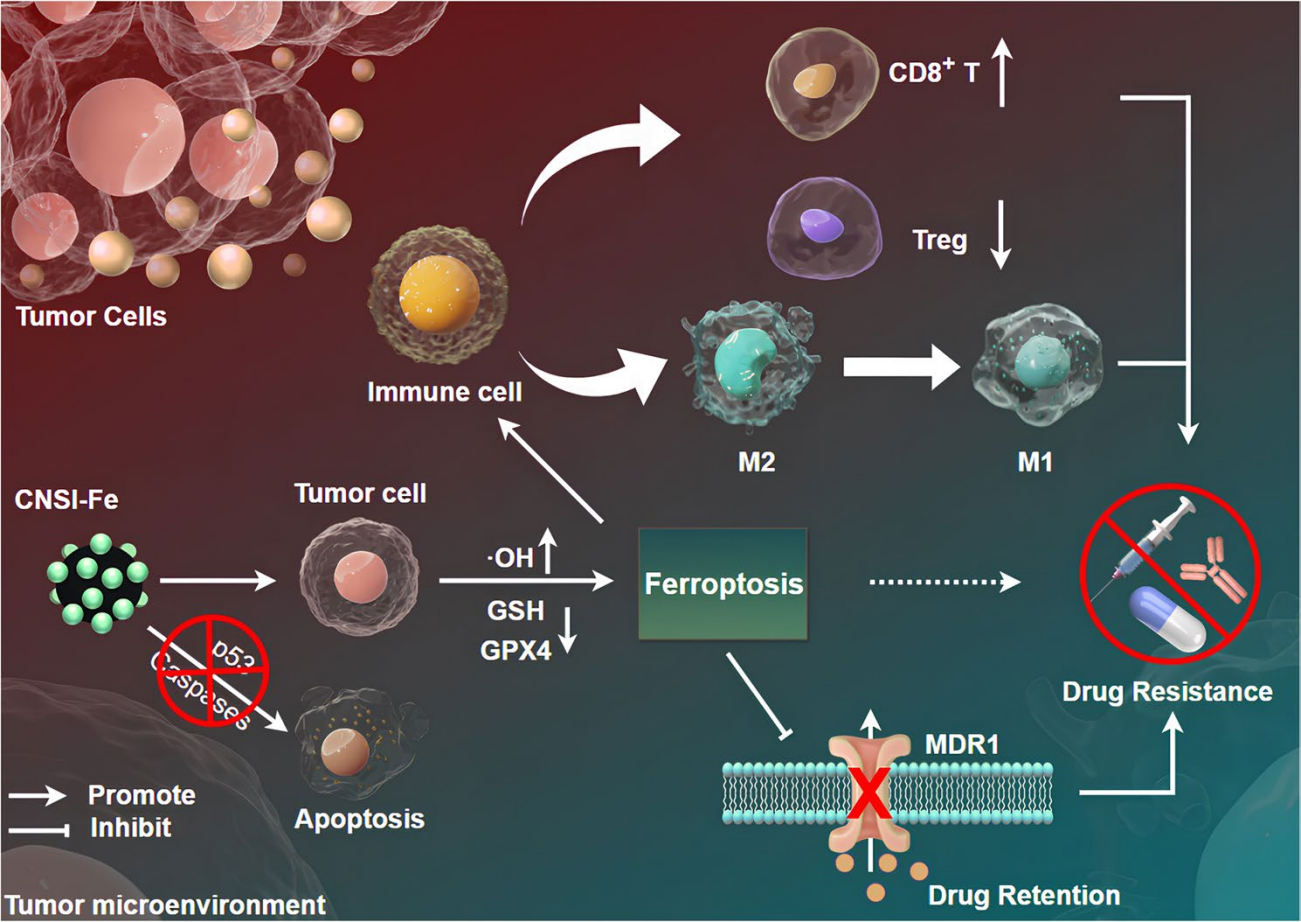
Schematic illustration of the mechanisms for therapy resensitization or hypersensitization by CNSI-Fe. p53, tumor protein p53; CD8^+^ T, cytotoxic T lymphocytes; Treg, regulatory T cells; M1, classically activated macrophages; M2, alternatively activated macrophages; •OH, hydroxyl radical; GSH, glutathione; GPX4, glutathione peroxidase 4; MDR1, multidrug resistance protein 1 (P-glycoprotein)

Preclinical evidence directly supported the possibility of CNSI-Fe-mediated effect: Naik et al. demonstrated that an iron(III)-bound nanocarbonaceous polyphenol (FeNCP) reverses chemoresistance by modulating intracellular glutathione pools and enhancing reactive oxygen species generation [33], providing a possible mechanistic basis for our observations. This phenomenon was also evident in patients with multiple metastases, where local injection into a single lesion resulted in systemic resensitization. This local-to-systemic effect is consistent with observations of intratumoral TLR agonists and oncolytic viruses [34, 35]. Considering the limited systemic activity of CNSI-Fe, this “local-to-systemic” effect suggested that the localized ferroptosis might functionally reset or attenuate the tumor’s MDR. Based on the existing literature and indirect observations, the following mechanisms might be likely involved (Fig. 3). (1) Inhibition of MDR transporters. Ferroptosis stress may inhibit the function of efflux transporters such as MDR1 or multidrug resistance-associated protein 1 (MRP1), leading to increased intracellular drug accumulation [36]. (2) Depletion of antioxidant defense system. Overwhelming uptake of Fe²⁺ leads to the accumulation of hydroxyl radicals and 4‑hydroxynonenal (4‑HNE), which in turn depletes glutathione peroxidase 4 (GPX4) and glutathione (GSH), creating a transient window of depleted antioxidant defense (Figure S8). This might enable the tumor to regain sensitivity to subsequent radiotherapy and chemotherapy, which involve oxidative damages [7]. (3) Bypassing apoptosis resistance. Ferroptosis does not rely on caspases or the p53 pathway. Therefore, even in tumor cells that have developed resistance to apoptosis or have multi-drug resistance, they remain sensitive to iron-induced lipid peroxidation [19]. (4) Reconstruction of tumor immune microenvironment [9]. CNSI-Fe-induced ferroptosis might polarize M2‑type macrophages toward M1 phenotype and promote CD8⁺ T cell infiltration. This process might reverse the immunosuppressive and therapy‑resistant microenvironment dominated by M2‑tpye macrophages [37]. Taken together, we hypothesized that these actions might collectively restore tumor sensitivity to subsequent therapies. This pattern of “re-empowering” subsequent therapies might likely serve as a potential driver for the survival outcomes that largely exceeded expectations. It should be noted that given the small sample size and the lack of mechanistic validation experiments in this exploratory discovery, the speculations still remain hypothetical. Future preclinical and translational studies are demanded to validate these mechanisms.

The potential impact of survivor bias must be carefully considered during interpreting the survival data [38, 39]. The four patients (4/19, 21.1%) who achieved long-term survival (> 28.9 months) had different primary tumor types (ovarian serous carcinoma, pancreatic adenocarcinoma, MELTVR, and papillary thyroid carcinoma). This cross-tumor distribution suggested that the survival advantage was unlikely to stem solely from the inclusion of a specific tumor type with a known favorable prognosis. However, these patients may represent a subgroup with more favorable tumor biology (e.g., heightened sensitivity to ferroptosis), although the generalizability of this finding requires validation in larger cohorts in future studies.

Beyond the encouraging phenomena, our study has its limitations, including small sample size (*n* = 19), lack of a control group, no patient-level ferroptosis biomarkers (e.g., GPX4, GSH, 4-HNE), tumor type heterogeneity (ovarian, pancreatic, thyroid, sarcoma, etc.), potential survivor bias, and unexcluded alternative explanations (e.g., natural disease variability, tumor heterogeneity, treatment sequencing). Generalizability is further limited by the intratumoral administration route, which requires accessible injectable lesions and may not be feasible for patients with diffuse metastases. In addition, the selected patient population (heavily pretreated, ECOG ≤ 1) may not represent broader solid tumor patients. These findings are hypothesis-generating and require validation in larger prospective studies in future evaluations.

## Conclusions

In summary, this exploratory analysis suggests that CNSI-Fe may be associated with signals of ferroptosis induction and treatment resistance reversal, as well as a potential survival benefit in a subset of patients with advanced, heavily pretreated solid tumors. However, these findings are hypothesis-generating due to the small sample size, single-arm design and speculative mechanisms. These preliminary observations warrant future expanded Ib/IIa clinical trials and phase II combination studies (with radiotherapy, chemotherapy, targeted therapy, or immunotherapy), as well as further mechanistic investigations into ferroptosis, immune microenvironment remodeling, and biomarker validation.

## Supplementary Information

Supplementary Material 1.

## Data Availability

The datasets used and/or analysed during the current study are available from the corresponding author on reasonable request.
