# Supplementary material for: First-in-human evidence of multidrug resistance reversal in solid tumors: a cohort analysis of carbon nanoparticles‑Fe(II) complex trials
Source: BMC Cancer. 2026 Jun 5;26:962. doi: 10.1186/s12885-026-16216-7 (PMC13459321; doi:10.1186/s12885-026-16216-7)
Supplement: Supplementary file 1 [file 12885_2026_16216_MOESM1_ESM.docx]

**Supplementary materials**

**1. Supplementary text**

**S1. Additional experimental protocols**

Nineteen patients received dose escalation following a “3+3” design, with sequential dose levels of 30, 60, 90, 120 and 150 mg (based on ferrous iron content). The drug was formulated as a sterile suspension at 30 or 60 mg/mL, with injection volumes ranging from 1 to 5 mL accordingly. Each cycle lasted for 21 days. On Day 1 of the first cycle, patients underwent intratumoral injection of CNSI-Fe (Table S1). If the investigator determined that continued treatment provided benefits outweighing the risks, the target lesion received a second intratumoral injection of the CNSI-Fe on Day 1 of the second cycle. All intratumoral injections were performed under ultrasound or CT guidance to ensure accurate drug delivery to the tumor site.

Safety and tolerability were assessed by monitoring adverse events (AEs), physical examination, vital signs, laboratory tests, Eastern Cooperative Oncology Group (ECOG) performance status, 12-lead electrocardiogram, and echocardiography. AEs were graded according to the Common Terminology Criteria for Adverse Events (CTCAE) version 5.0. The incidence of dose-limiting toxicities (DLTs) was evaluated to determine the maximum tolerated dose (MTD).

For CT imaging, treatment comprised 1 to 2 cycles of CNSI-Fe. For all patients, the first tumor assessment was scheduled 3 to 4 weeks after the initial cycle. Patients who received a second cycle underwent an additional assessment at 7 to 8 weeks post-first cycle. If the tumor assessment indicated a partial response (PR) or complete response (CR), an additional imaging assessment was conducted 4 weeks later for confirmation.

Based on imaging findings, the investigators will evaluate the antitumor efficacy of CNSI-Fe, for both the injected lesions and the overall tumor burden, with reference to the revised RECIST v1.1 and RECIST v1.1 criteria.

**S2. Additional description for Tables S2&S3**

A total of 10 patients survived for 12 months following CNSI-Fe treatment. Excluding the 4 resensitized/hypersensitized patients, who achieved a 24-month survival, the prior treatment details of the remaining 15 patients are provided in Table S2. The treatment sensitivity profiles to subsequent therapies following CNSI-Fe administration are presented in Table S3.

**2. Supplementary tables**

**Table S1.** Individual patient dosing information and number of treatment cycles.

| Number | Dose (mg) | Cycles | Dosing interval (d) |
| --- | --- | --- | --- |
| T01001 | 30 | 1 | / |
| T01002 | 30 | 1 | / |
| T01003 | 30 | 2 | 22 |
| T01004 | 60 | 2 | 25 |
| T01005 | 60 | 2 | 21 |
| T02001 | 60 | 1 | / |
| T02002 | 60 | 1 | / |
| T01006 | 90 | 1 | / |
| T01007 | 90 | 2 | 22 |
| T01009 | 90 | 2 | 34 |
| T01010 | 90 | 2 | 27 |
| T01011 | 90 | 1 | / |
| T03001 | 90 | 1 | / |
| T01012 | 120 | 1 | / |
| T01013 | 120 | 1 | / |
| T01014 | 120 | 2 | 27 |
| T01015 | 150 | 1 | / |
| T01016 | 150 | 1 | / |
| T01017 | 150 | 1 | / |

Note: /: No data.

**Table S2.** Baseline characteristics and prior therapies of the remaining 15 patients.

| Number | Tumor type | Stage | Prior therapies and therapeutic effects | | |
| --- | --- | --- | --- | --- | --- |
|  |  |  | Time before enrollment (month) | Treatment | Efficacy |
| T03001 | Fasciculated sarcoma | IV | ≈108 | Surgical resection^*^ | / |
|  |  |  | 68^+^ | Surgical resection^*^ | / |
|  |  |  | 55^+^ | Surgical resection^*^ | / |
|  |  |  | 43^+^ | Surgical resection^*^ | / |
|  |  |  | 37^+^ | Surgical resection^#^ | / |
|  |  |  | 37^+^ | Doxorubicin + Ifosfamide | / |
|  |  |  | 31^+^ | Radiotherapy | / |
|  |  |  | ≈20 | PD-1 antibody | PD |
| T01009 | Squamous cell carcinoma of the buccal mucosa | IV | 26^+^ | Radiotherapy | / |
|  |  |  | 25^+^ | Cisplatin | / |
|  |  |  | 4^+^ | Cetuximab + Carboplatin + Fluorouracil | / |
|  |  |  | 3^+^ | Cetuximab | PD |
| T01012 | Squamous cell carcinoma of the nasopharynx | IV | 44^+^ | Paclitaxel (albumin-bound type) + Cisplatin | / |
|  |  |  | 42^+^ | Radiotherapy + Nedaplatin | / |
|  |  |  | 41^+^ | Cisplatin | / |
|  |  |  | ≈22 | Clinical trial | PD |
|  |  |  | 5^+^ | Clinical trial | PD |
| T01014 | Adenocarcinoma of the right nasal cavity | IV | ≈225 | Surgical resection^*^ | / |
|  |  |  | ≈225 | Radiotherapy | / |
|  |  |  | 78^+^ | Surgical resection^*^ | / |
|  |  |  | 78^+^ | Radiotherapy | / |
|  |  |  | 78^+^ | Docetaxel + Nedaplatin | / |
|  |  |  | 40^+^ | Paclitaxel + Carboplatin + Tislelizumab | / |
|  |  |  | ≈35 | Tislelizumab | PD |
|  |  |  | ≈30 | Surgical resection^*^ | / |
|  |  |  | ≈12 | Radiotherapy | PD |
| T01016 | Breast carcinoma | IV | 125^+^ | Surgical resection^*^ | / |
|  |  |  | ≈125 | Taxane + Cyclophosphamide (TC) | / |
|  |  |  | ≈122 | Tamoxifen tablets | / |
|  |  |  | 98^+^ | Surgical resection^*^ | / |
|  |  |  | ≈98 | Paclitaxel + Carboplatin + Trastuzumab | / |
|  |  |  | ≈93 | Trastuzumab | / |
|  |  |  | 38^+^ | Surgical resection^*^ | / |
|  |  |  | 38^+^ | Doxorubicin + Cyclophosphamide + Taxane (AC-T) | / |
|  |  |  | 22^+^ | Albumin paclitaxel + Capecitabine | / |
|  |  |  | 15^+^ | Gemcitabine + Radiotherapy | / |
|  |  |  | 12^+^ | Elibrin + Anlotinib | PD |
| T01015 | Lung adenocarcinoma | IV | 100^+^ | Surgical resection^*^ | / |
|  |  |  | ≈100 | Icotinib | PD |
|  |  |  | 55^+^ | Pemetrexed + Cisplatin + Sintilimab or Placebo + Bevacizumab Biosimilar or Placebo | PR |
|  |  |  | 53^+^ | Pemetrexed + Cisplatin + Sintilimab or Placebo | / |
|  |  |  | 52^+^ | Pemetrexed + Sintilimab or Placebo | PD |
|  |  |  | 26^+^ | Docetaxel + Carboplatin + Bevacizumab | / |
|  |  |  | 23^+^ | Docetaxel + Carboplatin | / |
|  |  |  | 19 | Docetaxel + Carboplatin + Bevacizumab | / |
|  |  |  | 16^+^ | Docetaxel | / |
|  |  |  | 15^+^ | Docetaxel + Carboplatin + Bevacizumab | / |
|  |  |  | 11^+^ | Bevacizumab | PD |
|  |  |  | 10^+^ | Paclitaxel (albumin-bound type) + Bevacizumab | PD |
|  |  |  | 5^+^ | Anlotinib | PD |
| T01004 | Breast lobular ductal carcinoma | IV | 53^+^ | Docetaxel + Pirarubicin + Cyclophosphamide | / |
|  |  |  | ≈38 | Aidi injection | / |
|  |  |  | 16^+^ | Fulvestrant injection | / |
|  |  |  | 13^+^ | Vinorelbine tartrate + Capecitabine | / |
|  |  |  | ≈13 | Abemaciclib tablets | / |
|  |  |  | ≈11 | Etoposide Capsules | / |
|  |  |  | 5^+^ | Irinotecan + Bevacizumab | / |
|  |  |  | 1^+^ | Bevacizumab + Kanglaite injection | PD |
| T01013 | Fasciculated sarcoma | IV | 11^+^ | Epirubicin + Ifosfamide + Lenvatinib | / |
|  |  |  | 9^+^ | Epirubicin + Ifosfamide + Anlotinib | PD |
|  |  |  | 4^+^ | Radiotherapy + Anlotinib | PD |
| T02001 | Vulvar squamous cell carcinoma | IV | 5^+^ | Radiotherapy + Cisplatin | / |
|  |  |  | 2^+^ | Albumin-bound paclitaxel + Cisplatin | PD |
| T01002 | Lung squamous cell carcinoma | IVB | ≈11 | Surgical resection* | PD |
|  |  |  | 9^+^ | Docetaxel + Cisplatin + Deep Heat Therapy | / |
|  |  |  | 8^+^ | Cedicatedinibumab + Gemcitabine + Cisplatin + Deep Heat Therapy | / |
|  |  |  | ≈4 | Anlotinib | PD |
| T01007 | Liposarcoma | IV | 67^+^ | Surgical resection* | / |
|  |  |  | 26^+^ | Surgical resection* | / |
|  |  |  | 25^+^ | Ifosfamide + Pirarubicin + Deep Heat Therapy | PD |
| T01011 | Liposarcoma | IV | 24^+^ | Surgical resection* | / |
|  |  |  | 15^+^ | Surgical resection* | / |
|  |  |  | ≈13 | Ifosfamide + Epirubicin + Panaxapril | / |
|  |  |  | 11^+^ | Radiotherapy | / |
|  |  |  | ≈5 | Pamipirone + Epirubicin | / |
|  |  |  | ≈4 | Panaxapril | PD |
|  |  |  | 3^+^ | Iodine-125 seed implantation procedure | PD |
| T01006 | Pancreatic carcinoma | III | 9^+^ | Surgical resection* | / |
|  |  |  | 5^+^ | Albumin-bound paclitaxel + Tegafur/Gimeracil/Oteracil Potassium | PD |
|  |  |  | 3^+^ | Oxaliplatin + Irinotecan + Calcium Folinate + Fluorouracil | PD |
| T01010 | Sarcoma of the left mandible | IIIB | 148^+^ | Surgical resection* + Radiotherapy | / |
|  |  |  | 35^+^ | Surgical resection* | / |
|  |  |  | 33^+^ | Epirubicin + Ifosfamide + Mesna | / |
|  |  |  | 8^+^ | Anlotinib | PD |
|  |  |  | 5^+^ | Epirubicin + Ifosfamide + Mesna | PD |
| T01017 | Lung adenocarcinoma | IVB | ≈22 | Alectinib | / |
|  |  |  | ≈21 | Paclitaxel + Carboplatin | / |
|  |  |  | ≈21 | Enshatinib | / |
|  |  |  | ≈15 | Alectinib | / |
|  |  |  | ≈14 | Lorlatinib | / |
|  |  |  | ≈11 | Paclitaxel + Carboplatin | / |
|  |  |  | ≈7 | Alectinib | / |
|  |  |  | 5^+^ | Docetaxel + Cisplatin | PD |

Note: *: Tumor Resection; #: Lymph Node Dissection; /: No data.

PD, progressive disease.

**Table S3**. Summary of disease control and resensitization/hypersensitization of the remaining 15 patients.

| Number | Tumor type | Efficacy* | Subsequent treatment and therapeutic effect | | | Resensitization/  Hypersensitization | OS (month) | Survive |
| --- | --- | --- | --- | --- | --- | --- | --- | --- |
|  |  |  | Time after first administration (month) | Treatment | Efficacy* |  |  |  |
| T03001 | Fasciculated sarcoma | SD (-23.0%) | ≈5 | Anlotinib | / | Hypersensitization | 23^+^ | No |
| T01009 | Squamous cell carcinoma of the buccal mucosa | SD (-14.6%) | 2^+^ | Chemotherapy + Radiotherapy | / | / | 21^+^ | Yes |
| T01012 | Squamous cell carcinoma of the nasopharynx | SD (+3.1%), SD (+10.2%) | ≈1 | Clinical trial (Targeted therapy) | PD | / | 17^+^ | Yes |
|  |  |  | / | Immune + Targeted therapy | / |  |  |  |
| T01014 | Adenocarcinoma of the right nasal cavity | PD (+30.0%) | ≈4 | Chemotherapy | / | / | 17^+^ | Yes |
| T01016 | Breast carcinoma | SD (-22.3%) | 8^+^ | Anlotinib + Eribulin + Immunotherapy | SD | / | 14^+^ | Yes |
|  |  |  | 11^+^ | Trastuzumab deruxtecan |  |  |  |  |
| T01015 | Lung adenocarcinoma | SD (-2.6%) | 2 | Targeted therapy | / | / | 14^+^ | No |
| T01004 | Breast lobular ductal carcinoma | SD (+3.4%) | / | / | / | / | 7^+^ | No |
| T01013 | Fasciculated sarcoma | SD (-1.3%) | / | / | / | / | 5^+^ | No |
| T02001 | Vulvar squamous cell carcinoma | PD (+25.5%) | / | Clinical trials | / | / | 5^+^ | No |
| T01002 | Lung squamous cell carcinoma | PD (+54.3%) | / | Untreated | / | / | 4^+^ | No |
| T01007 | Liposarcoma | SD (+10.9%) | / | Untreated | / | / | 4^+^ | No |
| T01011 | Liposarcoma | SD (+19.5%) | / | Antitumor therapy | / | / | 4^+^ | No |
| T01006 | Pancreatic carcinoma | Not evaluated | / | Untreated | / | / | 2^+^ | No |
| T01010 | Sarcoma of the left mandible | SD (-6.2%) | / | / | / | / | 1^+^ | No |
| T01017 | Lung adenocarcinoma | SD (+7.8%) | / | / | / | / | 1^+^ | No |

Note: *: Efficacy of the injected lesion; /: No data.

OS, overall survival; SD, stable disease; PD, progressive disease.

**3. Supplementary figures**

**Figure S1.** Distribution of pre- and post-treatment TGR in 14 patients.

**Figure S2.** Comparison of survival time between patients with PD and patients with SD/CR based on overall efficacy at treatment discontinuation.

At the time of treatment discontinuation, the overall efficacy assessment included 6 patients with PD, 11 with SD, 1 with CR, and 1 patient who was not evaluable for efficacy, resulting in a total of 18 evaluable patients. Among patients assessed as PD, the mean OS was 7.2 months (95% CI: 2.9–11.6 months), the median OS was 5.2 months (95% CI: 3.6-6.8 months), the 1‑year OS rate was 20.0%, and the 2‑year OS rate was not reached. Among patients assessed as SD/CR, the mean OS was 26.9 months (95% CI: 19.3–34.4 months), the median OS was not reached, the 1‑year and 2‑year OS rates was 82.5% and 58.7%, respectively. Survival time differed significantly between patients with PD and those with SD/CR at treatment discontinuation (*p* = 0.016), with patients in the SD/CR group showing significantly prolonged survival.

**Figure S3.** Comparison of survival time between patients with the largest target lesion <5 cm and patients with the largest target lesion >5 cm.

Of the 19 patients who received the treatment, 11 had a largest target lesion <5 cm, and 8 had a largest target lesion >5 cm. Among patients with a largest target lesion <5 cm, the mean OS was 28.5 months (95% CI: 21.0–36.0 months), the median OS was not reached, and the 1‑year and 2‑year OS rates were 81.8% and 65.5%, respectively. Among patients with a largest target lesion >5 cm, the mean OS was 6.3 months (95% CI: 2.7–9.9 months), the median OS was 5.2 months (95% CI: 3.7–6.7 months), and the 1‑year survival rate was 19.0%. Survival time differed significantly between patients with a largest target lesion <5 cm and those with a largest target lesion >5 cm (*p* = 0.001), with patients in the <5 cm group showing significantly prolonged survival.

**Figure S4.** Comparison of survival time between patients with injection lesion <5 cm and patients with injection lesion >5 cm.

Of the 19 patients who received the treatment, 12 had an injected lesion <5 cm, and 7 had an injected lesion >5 cm. Among patients with an injected lesion <5 cm, the mean OS was 27.0 months (95% CI: 19.0–34.3 months), the median OS was not reached, and the 1‑year and 2‑year OS rates were 75.0% and 60.0%, respectively. Among patients with an injected lesion >5 cm, the mean OS was 6.3 months (95% CI: 1.8–10.9 months), the median OS was 4.4 months (95% CI: 2.2–6.7 months), and the 1‑year survival rate was 25.0%. Survival time differed significantly between patients with an injected lesion <5 cm and those with an injected lesion >5 cm (*p* = 0.002), with patients in the <5 cm group showing significantly prolonged survival.

**Figure S5.** Comparison of survival time between patients with target-only lesions and patients with both target and non-target lesions.

Among the 19 patients, 5 had target lesions only, while 14 had both target and non-target lesions. For patients with target lesions only, the mean OS was 23.9 months (95% CI: 14.1–33.7 months), the median OS was not reached, and the 1‑year and 2‑year OS rates were 80% and 53.3%, respectively. For patients with both target and non-target lesions, the mean OS was 19.0 months (95% CI: 10.5–27.5 months), the median OS was 14.1 months (95% CI: 0.0–28.7 months), and the 1‑year and 2‑year OS rates were 51.3% and 42.7%, respectively. Patients with target lesions only showed a trend toward prolonged survival. However, due to the small sample size, further studies with larger cohorts are warranted.

**Figure S6.** Comparison of survival time between patients with a single lesion and patients with multiple lesions.

Among the 19 patients, 4 had a single lesion, while 15 had multiple lesions (≥2 lesions). Among patients with a single lesion, the mean OS was 24.5 months (95% CI: 12.3–36.8 months), the median OS was not reached, and both the 1‑year and 2‑year OS rates were 75.0%. Among patients with multiple lesions (≥2 lesions), the mean OS was 18.4 months (95% CI: 10.6–26.2 months), the median OS was 14.1 months (95% CI: 0.0–33.4months), and the 1‑year and 2‑year OS rates were 55.0% and 31.4%, respectively. Patients with a single lesion showed a trend toward prolonged survival. However, due to the small sample size, further studies with larger cohorts are warranted.

**Figure S7.** Comparison of survival time between patients with ≤3 lesions and patients with >3 lesions.

Among the 19 patients, 9 had ≤3 lesions, and 10 had >3 lesions. Among patients with ≤3 lesions, the mean OS was 23.7 months (95% CI: 15.8–31.5 months), the median OS was not reached, and the 1‑year and 2‑year OS rates were 76.2% and 57.1%, respectively. Among patients with >3 lesions, the mean OS was 16.2 months (95% CI: 6.7–25.7 months), the median OS was 7.1 months (95% CI: 1.5–12.7 months), and the 1‑year and 2‑year OS rates were 44.4% and 33.3%, respectively. Patients with ≤3 lesions showed a trend toward prolonged survival. However, due to the small sample size, further studies with larger cohorts are warranted.

**Figure S8.** Schematic illustration of the mechanisms for ferroptosis induced by CNSI-Fe. DMT1, divalent metal transporter 1; FPN, ferroportin; TFR1, transferrin receptor 1; STEAP3, six-transmembrane epithelial antigen of prostate 3; NADP+, nicotinamide adenine dinucleotide phosphate (oxidized form); NADPH, nicotinamide adenine dinucleotide phosphate (reduced form); FSP1, ferroptosis suppressor protein 1; CoQ, coenzyme Q; CoQH2, coenzyme Q (reduced form, ubiquinol); H_2_O_2_, hydrogen peroxide; •OH, hydroxyl radical; SLC3A2, solute carrier family 3 member 2; SLC7A11, solute carrier family 7 member 11; GSSG, glutathione disulfide (oxidized form); GSH, glutathione (reduced form); GPX4, glutathione peroxidase 4; RSL3, Ras-selective lethal small molecule 3; PL-PUFA-OOH, phospholipid-hydroperoxide polyunsaturated fatty acid; PUFA, polyunsaturated fatty acid; ACSL4, acyl-CoA synthetase long-chain family member 4; PUFA-CoA, polyunsaturated fatty acid coenzyme A; LPCAT3, lysophosphatidylcholine acyltransferase 3; PL-PUFA, phospholipid-polyunsaturated fatty acid; POR, cytochrome P450 oxidoreductase; LOX, lipoxygenase; O_2_, oxygen; DHODH, dihydroorotate dehydrogenase.
